# Isoniazid Mono-Resistant Tuberculosis Presenting as Empyema Thoracis With Citrobacter koseri and Morganella morganii Infections: The World’s First Reported Case of Its Type

**DOI:** 10.7759/cureus.42767

**Published:** 2023-07-31

**Authors:** Sankalp Yadav

**Affiliations:** 1 Medicine, Shri Madan Lal Khurana Chest Clinic, New Delhi, IND

**Keywords:** morganella morganii, mtb (mycobacterium tuberculosis), citrobacter koseri, empyema thoracis, isoniazid mono-resistant tuberculosis

## Abstract

Drug resistance is very common in developing countries. Isolated cases of concomitant infection with *Mycobacterium tuberculosis*, *Citrobacter koseri*, and *Morganella morganii *are rare. Furthermore, there is no report available in the literature of concurrent infection of *Citrobacter koseri *and *Morganella morganii *in an isoniazid mono-resistant tuberculosis patient. In this case, we present a concomitant infection with drug-resistant strains of *Mycobacterium tuberculosis*, *Citrobacter koseri*, and *Morganella morganii *in a 40-year-old Indian male who presented with fever, dry cough, and chest pain. He was initiated on an isoniazid mono regimen and a broad-spectrum antibiotic, following the national guidelines.

## Introduction

Bacterial infections are prevalent in highly populated countries [1]. *Mycobacterium tuberculosis *is especially common in endemic settings [2], posing a significant threat to public health systems [3]. According to the India Tuberculosis Report 2023, there has been a substantial increase in the notification of drug-resistant tuberculosis cases [4]. Data from 2022 indicates that India reported 15,953 cases of isoniazid mono- or poly-resistant tuberculosis [3,4].

*Citrobacter koseri* belongs to the Enterobacteriaceae family, which comprises 11 different species, and is commonly found in water, soil, as well as the gastrointestinal tracts of humans and animals [5]. Infections with this bacterium are typically observed in patients with other comorbidities and are infrequent in the general population [5].

Similarly, *Morganella morganii* is a gram-negative rod primarily found in the environment and serves as normal flora in the intestines of humans, mammals, and reptiles [6]. It rarely causes community-acquired infections and is typically associated with postoperative and other hospital settings [7].

A case of concomitant infection with drug-resistant strains of *Mycobacterium tuberculosis*, *Citrobacter koseri*, and *Morganella morganii* is presented in a 40-year-old Indian male who was initiated on appropriate anti-tubercular chemotherapy and broad-spectrum antibiotics per the national guidelines.

## Case presentation

In the year 2019, a 40-year-old Indian male, who worked as a laborer and belonged to a low socioeconomic status, presented with complaints of fever, dry cough, and right-sided chest pain. He had experienced a low-grade fever 27 days prior to his visit that would rise in the evening, but it was not accompanied by chills or rigor and would subside after taking an over-the-counter antipyretic. Additionally, he had been experiencing a dry cough for 16 days and right-sided chest pain for 15 days. Initially, the chest pain occurred intermittently, but over the last 12 days, it has become more frequent with movement and is partially relieved when he rests.

There were no hemoptysis, seizures, weight loss, or night sweats. The patient had a history of drug-sensitive tuberculosis 28 years ago, during which he was treated for left cervical lymphadenitis by a private practitioner for three months, but no further details were available. He completed the treatment and had no relapses. Additionally, there was no history of substance abuse, imprisonment, or staying at a night shelter. Moreover, there was no history of diabetes, hypertension, or blood transfusions.

A general examination revealed an average-built man with a pulse rate of 76 beats per minute, a blood pressure of 110/76 mmHg, a temperature of 98.4 °F, an oxygen saturation of 99% in room air, and a respiratory rate of 17 breaths per minute.

Systemic examination indicated a dull note on percussion on the right lung with reduced breath sounds and decreased tactile and vocal fremitus on auscultation. The remainder of the systemic examination was within normal limits.

He underwent a routine diagnostic workup, which included induced sputum fluorescent microscopy (rhodamine auramine staining for *Mycobacterium tuberculosis*), a chest radiograph, a cartridge-based nucleic acid amplification test of the induced sputum, an electrocardiogram, and an ultrasound of the chest. His electrocardiogram was found to be normal.

**Figure 1:**
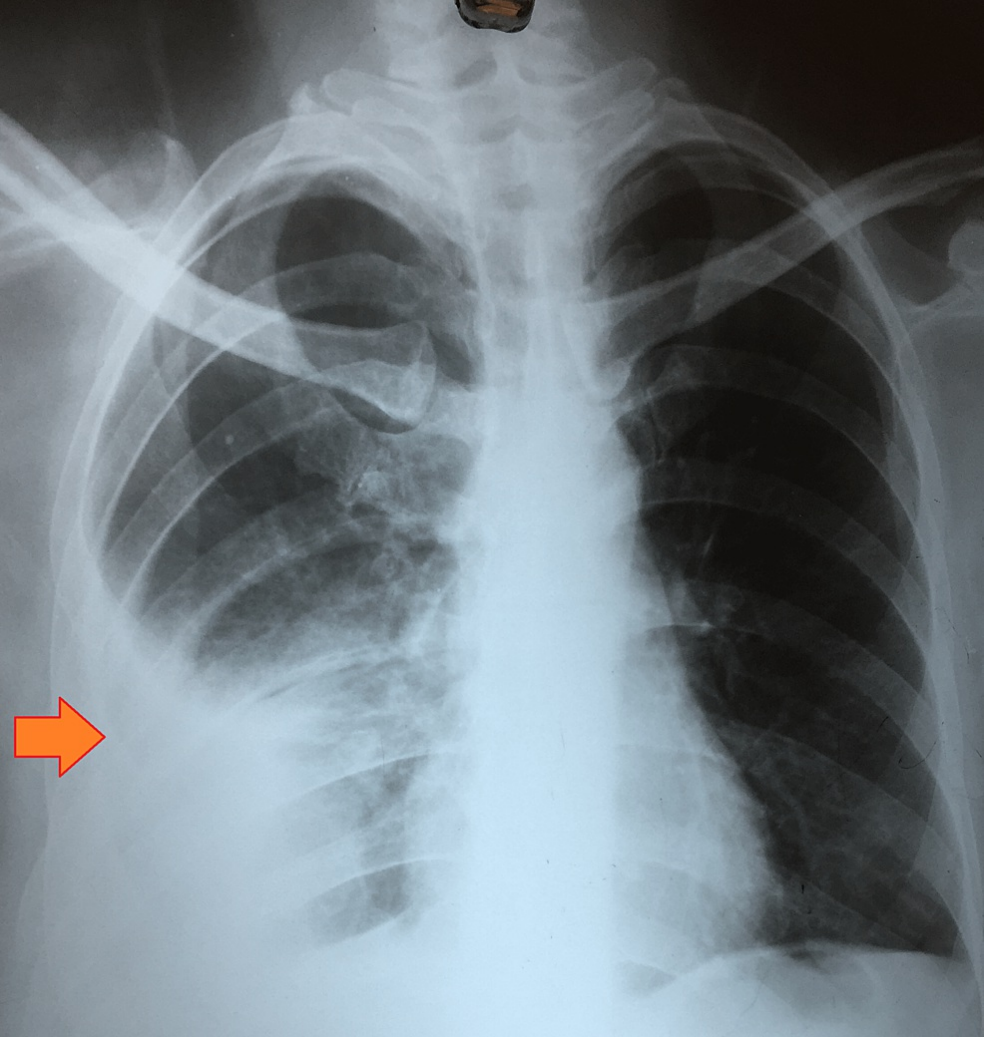
A chest radiograph (P-A) view suggestive of right-sided pleural effusion with consolidation in the right middle lobe P-A: postero-anterior

The induced sputum fluorescent microscopy and cartridge-based nucleic acid amplification test both yielded negative results. However, the chest radiograph showed evidence of right-sided pleural effusion with consolidation in the right middle lobe (Figure 1).

The laboratory workup revealed a hemoglobin level of 11.1 g/dL and an elevated erythrocyte sedimentation rate of 49 mm in the first hour. The hepatitis panel (hepatitis A, B, and C) and human immunodeficiency virus (I and II) tests were negative. Both blood and urine cultures showed negative results. All other routine laboratory parameters were within normal limits.

**Figure 2:**
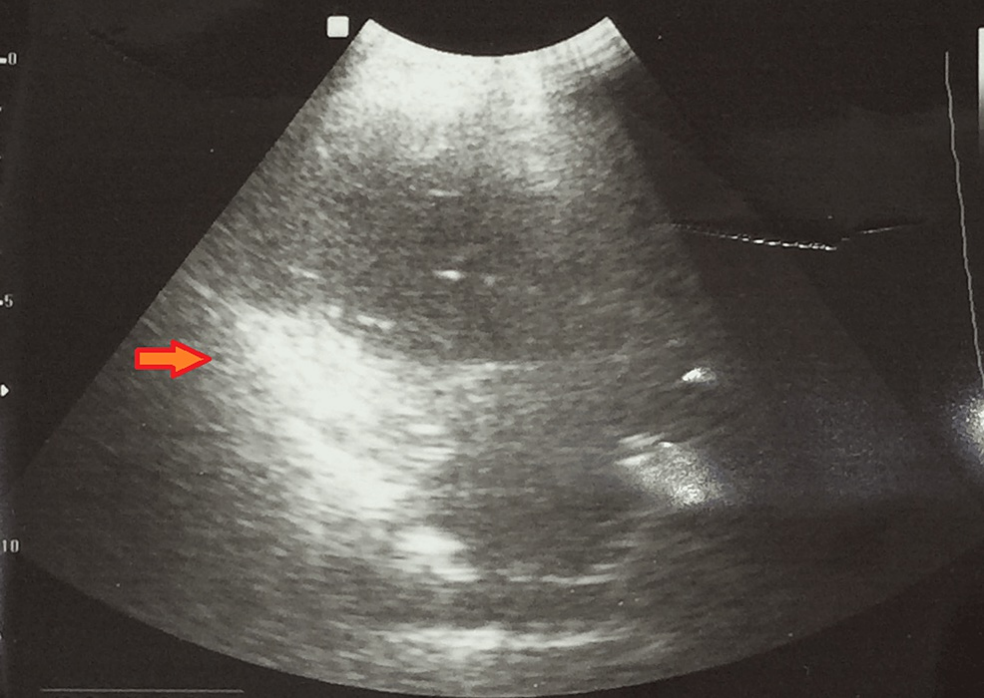
An ultrasound of the chest showing right-sided pleural effusion

An ultrasound of the chest showed a notable pleural effusion measuring 60.8 × 49.1 × 11.5 mm in size (approximately 26-29 cc). Additionally, there was pleural thickening on the right middle and lower hemithorax measuring 2.16 cm with low-level echoes (Figure 2).

He was referred to a higher center for diagnostic pleural tapping. A contrast-enhanced computed tomography of the chest indicated a right-sided encysted pleural effusion with consolidation in the right middle lobe. Consequently, intercostal drainage was performed, followed by intrapleural fibrinolysis. The pleural fluid collected was exudate in nature, showing high protein, low sugar, and a predominance of lymphocytes. However, Ziehl-Neelsen staining for acid-fast bacilli, cartridge-based nucleic acid amplification tests, and Gram staining yielded negative results. Nevertheless, the line probe assay suggested the presence of *Mycobacterium tuberculosis *with isoniazid mono-resistance (high level on the katG gene).

After four days, his intercostal drainage was removed, and suturing was performed. The sutures were removed on the tenth day. The pus culture results indicated the growth of *Mycobacterium tuberculosis *with isoniazid mono-resistance. Additionally, the culture identified *Citrobacter koseri *and *Morganella morganii*; the results of drug susceptibility testing are provided in Table 1.

**Table 1:** Drug susceptibility testing results

Isolate	Sensitive	Resistant
Mycobacterium tuberculosis	Rifampicin, ethambutol, pyrazinamide, kanamycin, moxifloaxacin (0.5), ofloxacin, levofloxacin	Isoniazid
Citrobacter koseri	Amikacin, gentamycin, meropenam, and piperacillin/tazobactam	Cefotaxime, ceftriaxone, ciprofloxacin, cefepime, tobramycin, ampicillin
Morganella morganii	Meropenam and piperacillin/tazobactam	Cefotaxime, ceftazidime, amikacin, ceftriaxone, gentamycin, ciprofloxacin, cefepime, tobramycin, and ampicillin

His pulmonary function test revealed a severely reduced vital capacity with no obstruction. A pleural biopsy was performed, and histopathology indicated a thickened pleura with non-specific chronic pleuritis and the presence of lymphoplasmacytic inflammatory cell infiltrates. A final diagnosis of isoniazid mono-resistant tuberculosis presenting as empyema thoracis with *Citrobacter koseri *and *Morganella morganii *infections was made. He was advised to undergo a pretreatment evaluation for the initiation of treatment following the national guidelines. The pre-treatment evaluation showed no abnormalities; therefore, he was started on the isoniazid-mono regimen (Table 2).

**Table 2:** Isoniazid-mono regimen per the national guidelines

Drug	Route of administration	Dose
Rifampicin	Per oral	450 mg
Ethambutol	Per oral	800 mg
Pyrazinamide	Per oral	1250 mg
Levofloxacin	Per oral	750 mg

**Figure 3:**
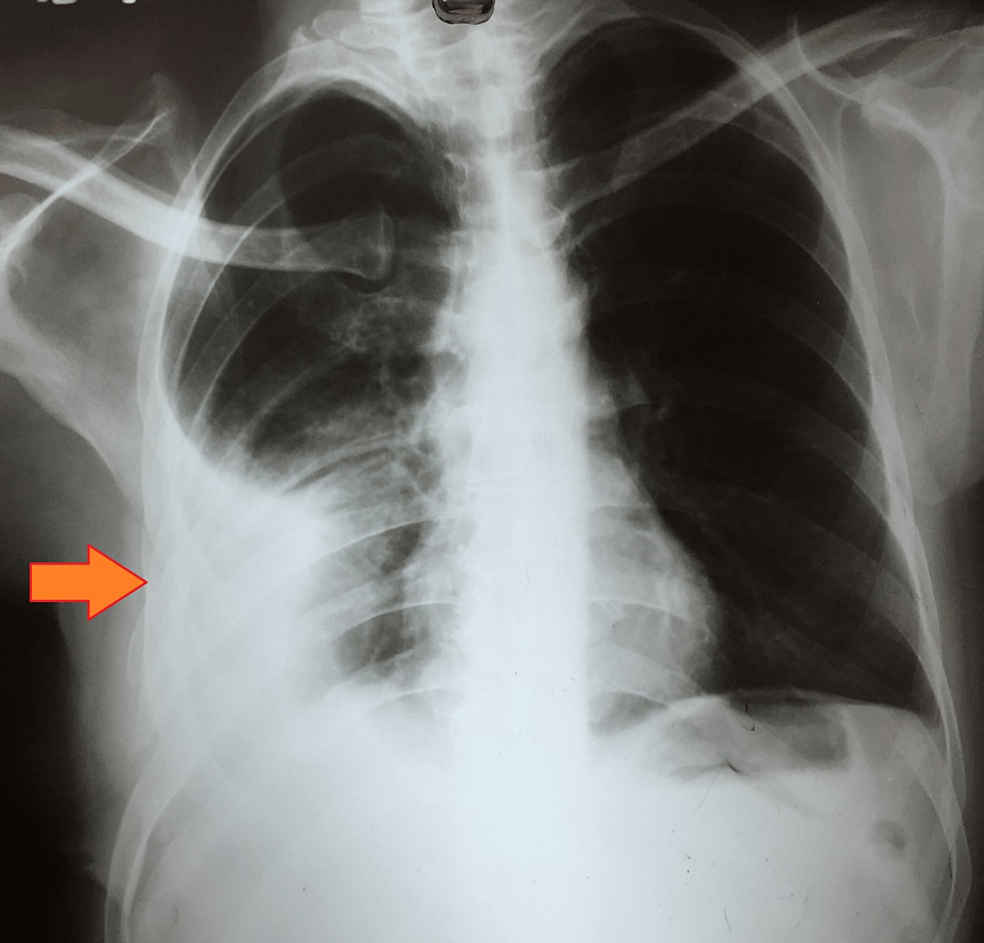
Chest radiograph (P-A) view at the completion of six months P-A: postero-anterior

For *Citrobacter koseri *and* Morganella morganii*, meropenam (1 g intravenous, 8 hourly) therapy was given for two weeks. The patient was also advised postural physiotherapy, incentive spirometry, and a high-protein diet. His chest radiograph at the sixth month is shown in Figure 3.

As there was incomplete resolution at the sixth month, his treatment was extended for another three months, and a repeat workup for drug resistance was planned. The smear microscopy results of induced sputum at the third, fourth, fifth, and sixth months were negative. However, he relocated to a different state and was eventually declared lost to follow-up.

## Discussion

Tuberculosis resistance to first-line anti-tubercular drugs poses a significant threat to public health measures [8]. Drug resistance can manifest as pre-extensively drug-resistant tuberculosis, which is resistant to rifampicin and any fluoroquinolone, isoniazid-resistant tuberculosis, rifampicin mono-resistant tuberculosis, multi-drug-resistant tuberculosis, or extensively drug-resistant tuberculosis, which is resistant to rifampicin plus any fluoroquinolone, plus a minimum of one of the drugs, such as bedaquiline and linezolid [9]. The most common form globally is isoniazid-resistant (sensitive to rifampicin), with a prevalence ranging from 0.0% to 9.5% [10].

*Citrobacter koseri *is a non-spore-forming, motile, Gram-negative bacillus that is a facultative anaerobe with the potential for aerobic respiration [11]. The most common site of infection is the abdominal cavity (51.1%), with other sites being the urinary tract (20%) and the lungs (11.1%) [11]. Further, it is documented that about 0.8% of Gram-negative bacterial infections were caused by *Citrobacter* species [12]. In humans, it results in neonatal meningitis and brain abscesses with high mortality rates [12]. A few cases of *Citrobacter koseri *causing severe infections or abscesses in adults have been reported [11].

*Morganella morganii* can result in thoracic empyema [13]. Usually, these infections are seen in immunocompromised hosts and are rare in immunocompetent cases [13]. However, with wider dissemination, an increase in antibiotic-resistant genes carried by the bacteria, and virulence evolution, it has become an important pathogen [7]. The infections caused by drug-resistant strains of *Morganella morganii *often lead to clinical treatment failure [7].

The diagnosis is difficult, especially due to the overlapping clinical features of pneumonia in *Mycobacterium tuberculosis*, *Citrobacter koseri*, and *Morganella morganii *infections. Also, community-acquired *Citrobacter koseri *and *Morganella morganii* infections are very rare, which delays the diagnosis [7]. However, in hospital settings, *Citrobacter koseri* infections account for about 3-6% of all Enterobacteriaceae infections [12]. In a study by Fischer et al., *Citrobacter koseri *and *Morganella morganii *infections were found to be 7.9% (9/114) [14].

A rare case of concomitant infection with drug-resistant strains of *Mycobacterium tuberculosis*, *Citrobacter koseri*, and *Morganella morganii* is presented here in a 40-year-old Indian male, which stresses the need for proactive efforts to rule out drug resistance even at presentation, especially in endemic countries. A detailed search of the literature showed that isolated primary isoniazid mono-resistant cases are available, but a case similar to the present one has never been reported.

In the end, this was only one case, and therefore it is recommended that similar presentations be reported, especially from high-burden countries. This will help not only in creating awareness about this rare condition but also in designing specific treatment regimens.

## Conclusions

A rare case of concomitant infection with drug-resistant strains of *Mycobacterium tuberculosis*, *Citrobacter koseri*, and *Morganella morganii* is presented in a 40-year-old Indian male. The clinical features overlapped, which resulted in a detailed laboratory and radiometric workup. Ultimately, a diagnosis was established and management was initiated after a discussion with an infectious disease expert, a microbiologist, and the drug-resistant tuberculosis committee per the national guidelines. This case stresses the need for raising awareness among primary care physicians about such rare presentations, as it requires a high degree of suspicion to diagnose and manage such cases.
